# Efficacy and Pharmacological Appropriateness of Cinnarizine and Dimenhydrinate in the Treatment of Vertigo and Related Symptoms

**DOI:** 10.3390/ijerph18094787

**Published:** 2021-04-30

**Authors:** Fulvio Plescia, Pietro Salvago, Francesco Dispenza, Giuseppe Messina, Emanuele Cannizzaro, Francesco Martines

**Affiliations:** 1 Department of Health Promotion Sciences Maternal and Infantile Care, Internal Medicine and Medical Specialities “Giuseppe D’Alessandro”, University of Palermo, Via del Vespro 133, 90127 Palermo, Italy; fulvio.plescia@unipa.it (F.P.); emanuele.cannizzaro@unipa.it (E.C.); 2 Dipartimento di Biomedicina, Neuroscienze e Diagnostica Avanzata (BiND), Sezione di Audiologia, Università Degli Studi di Palermo, Via del Vespro 129, 90127 Palermo, Italy; pietro.salvago01@unipa.it; 3 UOC Otorinolaringoiatria, A.O.U.P. “Paolo Giaccone”, Via del Vespro 129, 90127 Palermo, Italy; francesco.dispenza@gmail.com; 4 Department of Psychological, Pedagogical and Human Movement Sciences, University of Palermo, Via Giovanni Pascoli 6, Palermo 90144, Italy; giuseppe.messina17@unipa.it; 5 PosturaLab Center, 90127 Palermo, Italy

**Keywords:** cinnarizine, dimenhydrinate, vertigo, dizziness, pharmacological treatment of vertigo

## Abstract

Vertigo is not itself a disease, but rather a symptom of various syndromes and disorders that jeopardize balance function, which is essential for daily activities. It is an abnormal sensation of motion that usually occurs in the absence of motion, or when a motion is sensed inaccurately. Due to the complexity of the etiopathogenesis of vertigo, many pharmacological treatments have been tested for efficacy on vertigo. Among these drugs, cinnarizine, usually given together with dimenhydrinate, appears to be the first-line pharmacotherapy for the management of vertigo and inner ear disorders. Based on these considerations, the present non-interventional study aimed to investigate the clinical efficacy and tolerability of a fixed combination of cinnarizine (20 mg) and dimenhydrinate (40 mg) in patients suffering from vertigo-related symptoms. To this end, we enrolled 120 adults—70 males, and 50 females—with an average age of 64 years. Before beginning pharmacological treatment, all patients were screened for the intensity of vertigo, dizziness, and concomitant symptoms through the Visual Scale of Dizziness Disorders and Dizziness Handicap Inventory scales. At the end of the anamnestic evaluation, patients received the fixed-dose combination of cinnarizine (20 mg) plus dimenhydrinate (40 mg) 3 times daily, for 60 days. The results of this study provide further insight regarding the efficacy of the fixed combination when used to reduce symptoms of vestibular vertigo of central and/or peripheral origin, after both the 15- and 60-day therapies. Independent of the type of vertigo, the fixed combination was able to reduce dizziness- and vertigo-associated symptoms in more than 75% of all patients treated, starting from 15 days of therapy, and improving 60 days after starting the therapy. Interestingly, we also found differences between male and female patients in the framework of the pharmacological effects of therapy. This study provides further details concerning the therapeutic efficacy of the fixed combination of cinnarizine and dimenhydrinate, and also focuses attention on the possibility that these drugs could act in a gender-specific manner, paving the way for further research.

## 1. Introduction

Vertigo is not itself a disease, but rather a symptom of various syndromes and disorders that compromise balance function, which is essential for daily activities. Dizziness, vertigo, and disequilibrium may be due to complex modifications in central and peripheral neural activity [1]. In particular, an impairment of the vestibular system usually results from an alteration of the release of neurotransmitters and neuromodulators, which in turn affects the processing of sensory information [2,3,4,5,6,7].

Various studies have reported that vertigo is the most common complaint in patients of all ages, with a higher prevalence in adults over 60 years of age, and that about 15–20% of adults experience vertigo and dizziness yearly [8,9,10]. Furthermore, the prevalence rate of diseases increases with age, resulting in a steady rise in incidence due to the global rise in general life expectancy [11,12,13].

The maintenance of equilibrium is finely regulated by the brain, which tunes all the sensorial stimuli provided by the vestibular, visual, and proprioceptive systems, as well as the cognitive system [14,15,16,17]. Sensory inputs are further integrated and modulated by the extrapyramidal system, limbic system, and cerebral cortex [18].

The vestibular system is complex, and is usually divided into peripheral (the inner ear and the vestibular portion of the eighth cranial nerve) and central (vestibular nuclei, the oculomotor nuclei, the vestibulo–ocular reflex tracts, the cerebellum, the brainstem reticular formation, the area postrema, and other components) compartments [19,20]. Acute damage to the vestibular system promotes the genesis of different symptoms associated with vertigo, such as postural imbalance, nausea, vomiting, sensorineural hearing loss, migraine, and tinnitus [16,17,21,22,23,24,25]. Based on these considerations, it is evident that vertigo is a highly disabling disorder that negatively affects the ability to cope with daily activities, especially in elderly patients.

Because injury or neurodegenerative processes are responsible for damage to the vestibular system, understanding the etiopathogenesis of vestibular disease might be difficult. Therefore, this disorder requires an accurate diagnosis, including a detailed neuro-otological examination, in order to plan an adequate clinical intervention and/or a specific pharmacological strategy [26,27,28].

The first approach to a patient with vertigo may involve the use of pharmacological agents during the early acute phase of symptom onset, in order to provide fast relief. The goal of drug treatment is to suppress vestibular sensory input. This is thought to reduce conflicting sensory inputs, control undesirable perception, and improve the quality of the patient’s life [29,30,31].

Due to the complexity of the etiopathogenesis of vertigo, many pharmacological treatments are targeted to control the activity of neurotransmitters, neuromodulators, and voltage-gated channels that play a pivotal role in the modulation of neuronal excitability. In particular, drugs from several broad categories have been tested for efficacy on vertigo, including calcium channel antagonists, antihistamines, diuretics, corticosteroids, antipsychotics, and other psychotherapeutic drugs [32,33,34,35,36,37]. These drugs have different mechanisms of action. They may reduce the strength of symptoms (e.g., vestibular suppressants) or modify the underlying disease processes that have led to the development of symptoms (e.g., calcium channel antagonists in the case of vestibular migraine). Moreover, many of these drugs, especially those with sedative effects, also have the ability to restore balance to patients with vestibular damage [38].

Among all of these drugs, cinnarizine appears to be the first-line pharmacotherapy for the management of vertigo and inner ear disorders. It may act on the peripheral vestibular system, blocking voltage-gated calcium channels, preventing calcium translocations across the vestibular air cells and, thus, regulating hair cell afferent vestibular transmission [39,40,41]. Through these actions, it promotes anti-vasoconstrictor activity and a reduction in the blood viscosity of the inner ear’s circulatory system [42]. Cinnarizine has been used in clinical research at different doses, from 15 mg thrice-daily to 150 mg/day; however, its recommended clinical dose for the treatment of different vestibular disorders varies between 25 mg thrice-daily and 75 mg once-daily, up to a maximum of 225 mg [43,44]. Today, cinnarizine is usually given together with dimenhydrinate, a drug that exerts anti-vertigo and anti-emetic effects, acting as histamine (H1) receptor antagonist and phosphodiesterase inhibitor in the vestibular nuclei and the vomiting center [45].

Considering the limited publications in the last two decades about the pharmacological activity of cinnarizine plus dimenhydrinate in the treatment of vertigo, the present non-interventional study aimed to investigate the clinical efficacy and tolerability of a fixed combination of cinnarizine (20 mg) and dimenhydrinate (40 mg) in patients suffering from vertigo-related symptoms.

## 2. Patients, Materials and Methods

This prospective, open-label, non-interventional study was conducted from June 2020 to December 2020. The study enrolled 120 adults—70 males and 50 females (M/F ratio = 1.4) (mean age: 64 years); the female patients were on average slightly older than the male patients (Table 1). The recruited patients came from light work environments such as office employment, senior executives, and other work without exposure to noise.

Almost one third of the patients had taken antivertigo drugs before being included in the study, and it can be assumed that, in most cases, the previous medical treatment had been discontinued due to poor efficacy, and was replaced by the combined preparation.

On the first visit, all patients underwent a careful medical history collection, which included differential diagnosis, duration of vertigo, prior medical treatment, concurrent disease—such as hearing loss, tinnitus, and/or headache—and concomitant medications. Each subject underwent a careful otological examination using micro-otoscopy in order to rule out external/middle ear disease. A bedside examination—including tests for spontaneous nystagmus, smooth pursuit, and saccade, as well as the head shaking test and the Romberg test—was performed. In addition, a detailed neurological examination, including assessment of the cranial nerve and cerebellar functions, manual muscle testing for power, and somatosensory assessments, was also performed.

Exclusion criteria were: aged younger than 18 years, history of acute unilateral vestibulopathy, benign paroxysmal positional vertigo (BPPV), vestibular migraine, definite Ménière’s disease, MRI-documented retrocochlear disease (e.g., schwannoma), and exposure to ototoxic drugs.

The intensity of vertigo and concomitant symptoms were assessed by the Visual Scale of Dizziness Disorders, a five-point verbal rating (or visual analogue) scale ranging from no vertigo (0) to rarely (1), occasionally (2), frequently (3), and continuously (4). The following symptoms and triggering factors were investigated: instability, staggering, tendency to fall, swaying, position, inclination, walking, getting up, and head movements.

To value the perceived severity of dizziness, its impact on life, and the effectiveness of therapy, all patients were invited to complete the Dizziness Handicap Inventory (DHI). This tool is a 25-item survey with a total score ranging between 0 and 100 points, divided into three subscales: the functional subscale (36 points), the emotional subscale (36 points), and the physical subscale (28 points). Each item has 3 potential answers, with “yes” assigned 4 points, “sometimes” 2 points, and “no” 0 points.

**Figure 1:**
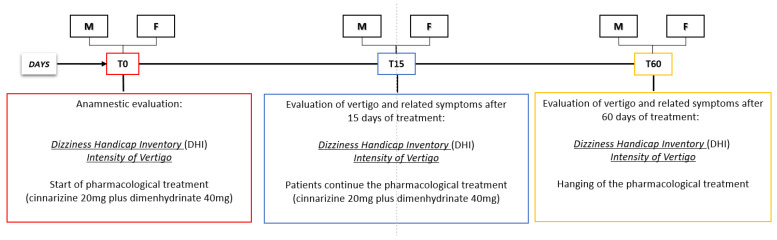
Timeline of the experimental procedures. M: male; F: female; T0: time at which the pharmacological treatment started; T15: 15 days after the beginning of the pharmacological treatment; T60: end of the treatment.

These parameters have been evaluated at the beginning of treatment (T0), 15 days after the treatment (T15), and at the end of the observational period (T60) (Figure 1). We chose a period of 60 days for the final evaluation of the drugs’ effects, because this is a useful period for vestibular restoration. At the end of the anamnestic evaluation, patients received the fixed-dose combination of cinnarizine (20 mg) plus dimenhydrinate (40 mg), 3 times daily for 60 days.

## 3. Statistical Analysis

Statistical analysis was performed using the GraphPad Prism 8.01 statistical software package (GraphPad Company, San Diego, CA, USA). Data were tested for normal distribution using the Kolmogorov–Smirnov test. Because of their normal distribution, the statistical analysis was performed using a parametric test, as reported.

Data obtained on the reduction of dizziness and vertigo symptoms were analyzed using a one-way ANOVA, followed by a post-hoc Tukey’s multiple comparison test. With regard to the data on the assessments of the vertiginous disorder, a two-way ANOVA was used, followed by a post-hoc Bonferroni test with ordinary alpha = 0.5. Data are reported as mean ± SD. Statistical significance was set at *p* < 0.05.

## 4. Results

### 4.1. Dizziness Handicap Inventory (DHI)

**Figure 2:**
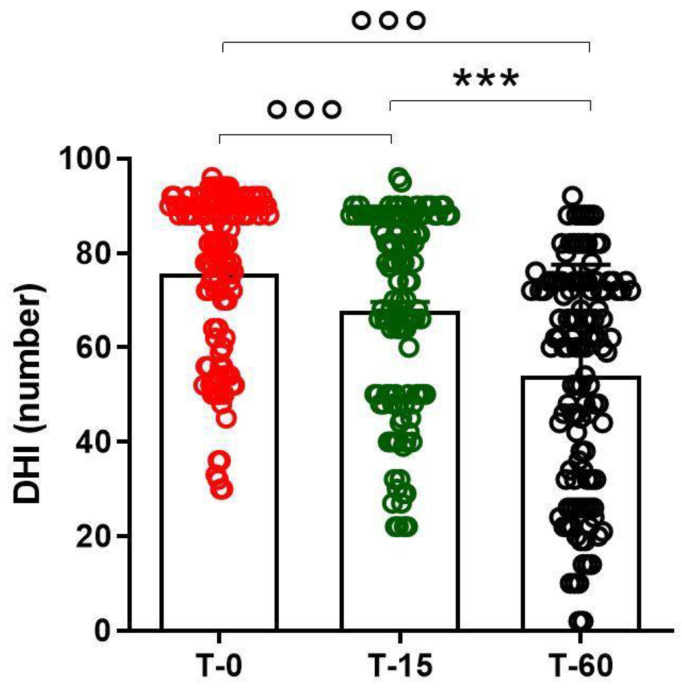
Reduction of dizziness symptoms, evaluated using the Dizziness Handicap Inventory (DHI), assessed at different times after pharmacological treatment with a fixed-combination preparation of cinnarizine (20 mg) plus dimenhydrinate (40 mg). Data refer to *n* = 120 patients, and are represented as the mean ± SD of DHI symptoms. *** *p* < 0.001 vs. T15; °°° *p* < 0.001 vs. T0. T0: baseline; T15: evaluation after 15 days from the start of therapy; T60: evaluation at the end of therapy.

The assessment of the effectiveness of pharmacological treatment on dizziness was conducted at three different points in time. In particular, statistical analysis conducted using a one-way ANOVA of the repeated measures performed to treat dizziness showed a significant effect of the treatment on all patients studied (F(2.119) = 154.6; *p* < 0.0001). When the data were analyzed so as to understand the time at which pharmacological treatment was most effective, a post-hoc Tukey’s multiple comparison test showed a significant reduction in dizziness symptoms at T60, when compared to T15 and T0 (*q* = 15.51 *p* < 0.001; *q* = 24.59 *p* < 0.001) and at T15 with respect to T0 (*q* = 9.082 *p* < 0.001) (Figure 2).

**Figure 3:**
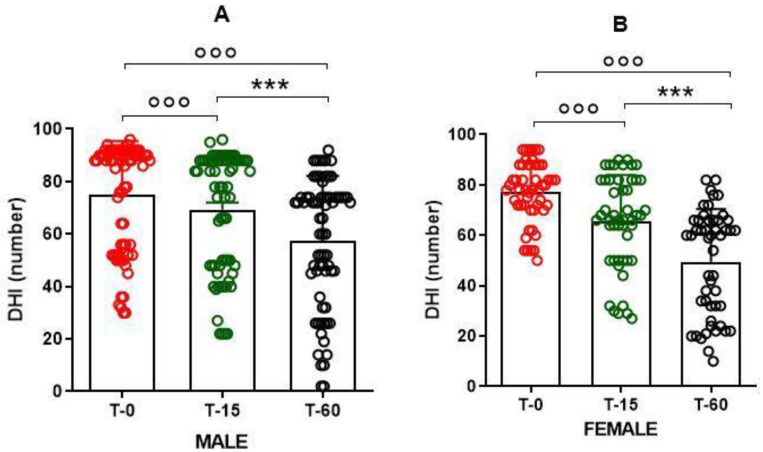
Reduction of dizziness symptoms, evaluated using the Dizziness Handicap Inventory (DHI), assessed at different times after pharmacological treatment with a fixed-combination preparation of cinnarizine (20 mg) plus dimenhydrinate (40 mg). Data refer to (**A**) *n* = 70 male, and (**B**) *n* = 50 female patients, and are represented as the mean ± SD of DHI symptoms. *** *p* < 0.001 vs. T15; °°° *p* < 0.001 vs. T0. T0: baseline; T15: evaluation after 15 days from the start of therapy; T60: evaluation at the end of therapy.

Furthermore, in order to evaluate whether pharmacological treatment was effective in both male and female patients, we also performed a statistical analysis by gender. A one-way ANOVA of the repeated measures performed to treat dizziness showed significant effects of the treatment on both male (F(2.69) = 21.81; *p* < 0.0001) and female (F(2.49) = 9.915; *p* < 0.0001) patients. Data from a post-hoc Tukey’s multiple comparison test showed a significant reduction in dizziness symptoms at T60, compared to to T15 and T0, in both male (*q* = 12.33 *p* < 0.001; *q* = 18.33 *p* < 0.001) and female (*q* = 10.48 *p* < 0.001; *q* = 17.97 *p* < 0.001) patients. We also found a significant reduction in dizziness symptoms from T15 onwards in both male (*q* = 5.677 *p* < 0.001) and female (*q* = 7.491 *p* < 0.001) patients (Figure 3A,B).

### 4.2. Vertigo Symptoms

#### 4.2.1. All Patients

**Figure 4:**
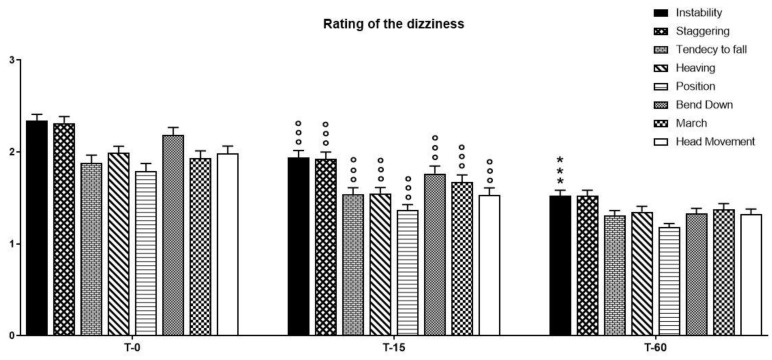
The intensity of vertigo and concomitant symptoms, as evaluated by the five-point verbal rating (or visual analogue) scale—ranging from no vertigo (0) to rarely (1), occasionally (2), frequently (3), and continuously (4)—has been taken into account in order to assess the efficacy of treatment with a fixed-combination preparation of cinnarizine (20 mg) plus dimenhydrinate (40 mg) on the reduction of the triggering factors of the dizziness itself. Data refer to *n* = 120 patients, and are represented as the mean ± SD of the factors able to trigger vertigo. *** *p* < 0.001 vs. T15; °°° *p* < 0.001 vs. T0. T0: baseline; T15: evaluation after 15 days from the start of therapy; T60: evaluation at the end of therapy.

Statistical analysis, performed based on the observations of the parameters obtained through the use of the Visual Scale of Dizziness Disorders on vegetative symptoms, highlighted a significant time effect of pharmacological treatment on the reduction of dizziness symptoms. A two-way ANOVA showed a significant effect of pharmacological treatment on time (F(2, 14) = 447.22, *p* < 0.0001), symptoms (F(7, 14) = 8.421, *p* < 0.0001), and their interaction (F(2, 7) = 4.169, *p* < 0.0001). A Bonferroni post-hoc analysis indicated that pharmacological treatment with a fixed-combination preparation of cinnarizine (20 mg) plus dimenhydrinate (40 mg) was able to reduce most of the symptoms related to the vertiginous disorder (Figure 4). In particular, a significant reduction in all analyzed symptoms was recorded 15 days after starting therapy (T15), compared to T0; furthermore, a reduction of instability was also detected between T0 and T60 (Table 2).

#### 4.2.2. Male Patients

**Figure 5:**
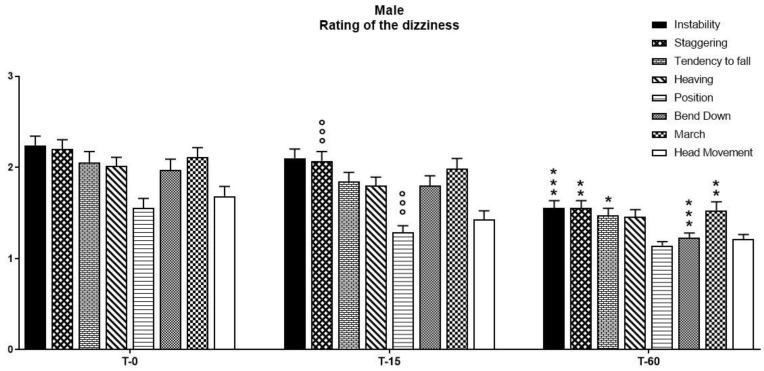
The intensity of vertigo and concomitant symptoms as evaluated by the five-point verbal rating (or visual analogue) scale—ranging from no vertigo (0) to rarely (1), occasionally (2), frequently (3), and continuously (4)—have been taken into account in order to assess the efficacy of treatment with a fixed-combination preparation of cinnarizine (20 mg) plus dimenhydrinate (40 mg) on the reduction of the triggering factors of the dizziness itself. Data refer to *n* = 70 male patients, and are represented as the mean ± SD of the factors able to trigger vertigo. * *p* < 0.05, ** *p* < 0.01, *** *p* < 0.001 vs. T15; °°° *p* < 0.001 vs. T0. T0: baseline; T15: evaluation after 15 days from the start of therapy; T60: evaluation at the end of therapy.

When statistical analysis was performed on the male patients, a two-way ANOVA performed on the observations of the parameters obtained through the use of the Visual Scale of Dizziness Disorders on vegetative symptoms revealed a significant effect of pharmacological treatment on time (F(2, 14) = 110.23, *p* < 0.0001), symptoms (F(7, 14) = 7.73, *p* < 0.0001), and their interaction (F(2, 7) = 8.90, *p* < 0.0001). A Bonferroni post-hoc analysis indicated that pharmacological treatment with a fixed-combination preparation of cinnarizine (20 mg) plus dimenhydrinate (40 mg) was able to reduce most of the symptoms related to the vertiginous disorder (Figure 5). In particular, a significant reduction in immobility, staggering, tendency to fall, bending down, and marching was recorded between T15 and T60, although a decrease in staggering and position was recorded at T15 compared to T0 (Table 3).

#### 4.2.3. Female Patients

**Figure 6:**
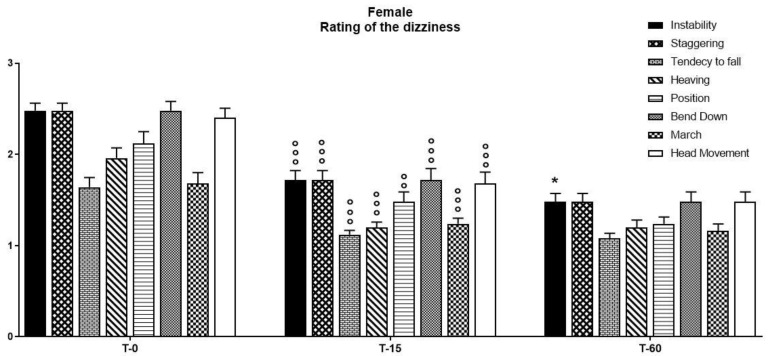
The intensity of vertigo and concomitant symptoms as evaluated by the five-point verbal rating (or visual analogue) scale—ranging from no vertigo (0) to rarely (1), occasionally (2), frequently (3), and continuously (4)—have been taken into account in order to assess the efficacy of treatment with a fixed-combination preparation of cinnarizine (20 mg) plus dimenhydrinate (40 mg) on the reduction of the triggering factors of the dizziness itself. Data refer to *n* = 50 female patients, and are represented as the mean ± SD of the factors able to trigger vertigo. * *p* < 0.05 vs. T15; °° *p* < 0.001, °°° *p* < 0.001 vs. T0. T0: baseline; T15: evaluation after 15 days from the start of therapy; T60: evaluation at the end of therapy.

When the data obtained was analyzed only for female subjects, a statistical analysis conducted using a two-way ANOVA performed on the observations of the parameters obtained through the use of the Visual Scale of Dizziness Disorders on vegetative symptoms highlighted a significant effect of time (F(2, 14) = 375.1, *p* < 0.0001), symptoms (F(7, 14) = 12.35, *p* < 0.0001), and their interaction (F(2, 7) = 12.35, *p* < 0.0001). In particular, a Bonferroni post-hoc analysis indicated that the pharmacological treatment with a fixed-combination preparation of cinnarizine (20 mg) plus dimenhydrinate (40 mg) was able to reduce the symptoms related to the vertiginous disorder (Figure 6). Interestingly, the reduction in all symptoms began after 15 days of therapy, and remained unchanged after 60 days of treatment (Table 4).

## 5. Discussion

The present non-interventional study investigated the pharmacological efficacy of a fixed low-dose combination of cinnarizine (20 mg) plus dimenhydrinate (40 mg), as used under real-life conditions in the treatment of dizziness- and vertigo-related symptoms. The study population consisted of a representative sample of patients with vertigo, observed daily in our medical practice. Specifically, concerning age and sex, patients who attended this study represented a typical vertigo population: mostly male, with a mean age of 64 years [46,47].

Although the complexity of the vestibular system and limited means make it difficult to establish an exact diagnosis, especially within the primary and secondary care settings, pharmacological management of patients with vertigo is of considerable importance, in particular for treating dizziness during the early acute phase of symptom onset [38,48].

The common objective of pharmacological treatment is to inhibit vestibular sensory input and restore blood flow by improving the microcirculation [49,50]. A wide variety of drugs is used to treat vertigo and its associated symptoms, such as nausea and emesis. These medications may influence the response of neurons by facilitating or inhibiting the effect of the primary neurotransmitters, or by altering patterns of release and thereby modifying sensory transmission in the vestibular sensory pathways.

In agreement with previous studies conducted by Pytel [51], Scholtz [52], and others, the results of this study provide further insight regarding the efficacy of the aforementioned fixed low-dose combination when used to reduce vestibular vertigo symptoms of central and/or peripheral origin, as measured by the Dizziness Handicap Inventory and the Visual Scale of Dizziness Disorders, after both the 15-day therapy (primary evaluation of the efficacy of pharmacological therapy) and 60-day therapy (primary efficacy endpoint). In particular, independent of the type of vertigo, the fixed-combination treatment was able to reduce dizziness- and vertigo-associated symptoms in more than 75% of all patients treated, starting after 15 days of therapy, and improving 60 days after starting the therapy, to the end of observation.

In general, positive pharmacological effects obtained in our study could be attributable to different actions of the two active principles contained in the preparation employed, which work synergistically to reduce dizziness and other related symptoms. Cinnarizine, acting on the peripheral vestibular system, can selectively inhibit spasmogen-stimulated Ca^2+^ influx and promote an anti-vasoconstrictor action [53] that consequently causes an increase in blood flow to the vestibular nuclei. This activity counters the insufficient cerebral blood circulation likely responsible for “vertiginous symptoms” such as dizziness, nausea, vomiting, and tinnitus [23,24,54,55]. Furthermore, dimenhydrinate, through the block of H1 receptors abundantly located throughout the central vestibular nuclear complex—including the medial vestibular nucleus—inhibits the spreading impulses at the medullar vestibular, which are closely associated with vegetative sensory regulation. This results in a decrease of the symptoms of vertigo and its associated vegetative complaints [7,56,57]. Thus, both active compounds act on structures embroiled in the pathogenesis of vertigo and associated vegetative symptoms.

In addition to evaluating the drugs’ effects on the total number of patients treated, we wanted to investigate whether this medical compound was equally effective on both male and female patients. Interestingly, when the efficacy of the drug was evaluated on female patients, the combined action of the two active principles has proved to be useful in improving and reversing all vertigo symptoms, reaching higher efficacy 15 days after starting therapy. Only instability symptoms tend to decrease further after 60 days of therapy. In contrast, in male patients, we observed a greater reduction only in some of the symptoms analyzed. By the 15th day after the beginning of therapy, only staggering- and position-associated dizziness symptoms tended to revert, and we had to wait 60 days before observing a reduction in many of the other examined symptoms.

The difference in drug response between male and female patients observed in our study may be due to sex-based differences that impact pharmacokinetics. It has been reported that sex-related differences could induce changes in terms of the absorption, distribution, metabolism, and elimination of drugs. This can differentially affect their efficacy and safety, explaining why some drugs may function much better in females than in males, or vice versa. This could guide changes in dosage regimens or therapeutic monitoring, so as to increase the drugs’ effectiveness or reduce potential toxicity [58,59]. Moreover, males and females are different in terms of physiology, and this can make a difference to the drugs’ response.

Among physiological parameters, it seems that sex hormones play a determining role in the different pharmacokinetic responses between males and females. In particular, they exert a huge influence on the dose, plasma levels, interval of administration, and excretion, with significant clinical effects that are especially important for drugs with narrow therapeutic indexes [60]. Furthermore, increased levels of estrogen and progesterone can alter hepatic enzyme activity, which can increase drug accumulation or decrease the elimination of some drugs.

Hormonal levels that change during the menstrual cycle, with the use of oral contraceptives, throughout pregnancy, or during menopause, may affect the metabolism of drugs, thus modifying their action [61,62]. Sex hormones are prominent in the processes that underly the expression of the cytochrome P450 mono-oxygenases (CYP)450 system [60,61,62,63,64,65].

On the other hand, it has been established that gender-related differences in the gene expression of drug-metabolizing enzymes introduce alterations in drug metabolism, possibly affecting drug efficacy and safety [66,67]. Furthermore, the biochemical basis of sex differences in drug metabolism was also shown to be related to hormonal regulation of the production of drug-metabolizing enzymes in animals and humans [68,69]. Inter-individual differences in drug metabolism are significantly influenced by polymorphisms in cytochrome P450 (CYP450). Variations in CYP450 activity may alter a drug’s efficacy and/or lead to the onset of adverse effects. Cinnarizine, and to a lesser extent dimenhydrinate, are metabolized by CYP2D6, an enzymatic system that exhibits a high genetic polymorphism, whose activity appears to be higher in men than in women [70,71,72]. The higher expression of CYP2D6 in males than in females may, in part, explain our results.

It is likely that the lower expression of CYP2D6 in females may promote a reduction in drug elimination and, consequently, increase the proportion of the administered drug that reaches the circulatory system. This potential increase might explain the increase in the drugs’ therapeutic effects beginning from 15 days after the first administration more in female than in male patients.

Concerning the tolerability of the study treatment, we did not find any particular adverse effects reported in other studies. A low-dose fixed combination was well tolerated by both sexes, and the only frequently reported adverse effects were drowsiness, sedation, amnesia, and headache.

There are some limitations to this study that should be considered in future research. For example, due to the limited sample size, it is difficult to generalize about the different effects of a fixed combination of cinnarizine (20 mg) and dimenhydrinate (40 mg) between males and females. Furthermore, the absence of a control group did not allow us to determine the real efficacy of the drug under study.

Understanding the correlation between gender differences in drug response could contribute to the implementation of more effective intervention and prevention strategies in repressing different diseases.

## 6. Conclusions

In conclusion, the present study provides further details concerning the potential therapeutic efficacy of the fixed combination of cinnarizine and dimenhydrinate that, given three times daily, has proven to be an effective and well-tolerated treatment to counteract central, peripheral, and combined central/peripheral vertigo. Therefore, our results reinforce and add value to what has been previously reported regarding the efficacy and appropriateness of prescribing cinnarizine and dimenhydrinate in vertigo care.

Furthermore, our results suggest that these drugs could act in a gender-specific manner, paving the way for further research. Understanding differences in the symptoms of vertigo disorders using gender-based approaches could improve pharmacological treatment and the quality of medical care.

## Figures and Tables

**Table 1:** Demographic data and other baseline characteristic of patients included in the study.

Parameters	Patients		Statistical Data				
	Na	%	Mean	SD	Range		
					Min	Med	Max
Age (years)			64.425	9.03	43	64	81
Male	70	55%	64.9	7.77	46	64	79
Female	50	45%	63.8	10.71	41	65	81
Height (cm)			1.61	0.08	1.45	1.6	1.75
Weight (kg)			71.49	12.1	42	72	95
BMI (Kg/m2)			26.45	3.71	17	26	31
Duration of Vertigo (month)			8	2.29	5	8	14

Na: Number; %: percentage of patients; Mean: mean value; SD: standard deviation; Range Min, Med, and Max, respectively: minimum, median and maximum.

**Table 2:** Statistical analysis obtained using a Bonferroni post-hoc test on the factors triggering the onset of vertigo.

**T0 Vs. T15**				
**Symptoms**	**Mean Difference**	***t***	***p* Value**	**Summary**
Instability	0.6100	6.801	*p* < 0.001	°°°
Staggering	0.9510	10.60	*p* < 0.001	°°°
Tendency to Fall	0.8550	9.533	*p* < 0.001	°°°
Heaving	0.4850	5.408	*p* < 0.001	°°°
Position	0.9500	10.59	*p* < 0.001	°°°
Bend Down	0.5950	6.634	*p* < 0.001	°°°
March	0.6350	7.080	*p* < 0.001	°°°
Head Movement	0.4900	5.463	*p* < 0.001	°°°
**T15 Vs. T60**				
**Symptoms**	**Mean Difference**	***t***	***p* Value**	**Summary**
Instability	0.5050	5.631	*p* < 0.001	***
Staggering	−0.1160	1.293	*p* > 0.05	ns
Tendency to Fall	0.2200	2.453	*p* > 0.05	ns
Heaving	0.1750	1.951	*p* > 0.05	ns
Position	0.1900	2.118	*p* > 0.05	ns
Bend Down	0.2350	2.620	*p* > 0.05	ns
March	0.2650	2.955	*p* > 0.05	ns
Head Movement	0.2050	2.286	*p* > 0.05	ns

Data refer to *n* = 120 patients. *** *p* < 0.001 vs. T15; °°° *p* < 0.001 vs. T0. T0: baseline; T15: evaluation after 15 days from the start of therapy; T60: evaluation at the end of therapy.

**Table 3:** Statistical analysis obtained using a Bonferroni post-hoc test on the factors triggering the onset of vertigo.

**T0 Vs. T15**				
**Symptoms**	**Mean Difference**	***t***	***p* Value**	**Summary**
Instability	−0.06000	0.4609	*p* > 0.05	ns
Staggering	1.142	8.773	*p* < 0.001	°°°
Tendency to Fall	0.2300	1.767	*p* > 0.05	ns
Heaving	0.2100	1.613	*p* > 0.05	ns
Position	1.260	9.680	*p* < 0.001	°°°
Bend Down	0.1100	0.8451	*p* > 0.05	ns
March	0.1100	0.8651	*p* > 0.05	ns
Head Movement	0.2600	1.997	*p* > 0.05	ns
**T15 Vs. T60**				
**Symptoms**	**Mean Difference**	***t***	***p* Value**	**Summary**
Instability	0.5300	4.072	*p* < 0.001	***
Staggering	−0.4720	3.626	*p* < 0.01	**
Tendency to Fall	0.4200	3.227	*p* < 0.05	*
Heaving	0.3500	2.689	*p* > 0.05	ns
Position	0.1400	1.076	*p* > 0.001	ns
Bend Down	0.5500	4.225	*p* < 0.001	***
March	0.4400	3.380	*p* < 0.01	**
Head Movement	0.2100	1.613	*p* > 0.05	ns

Data refer to n = 70 male patients. * *p* < 0.05, ** *p* < 0.01, *** *p* < 0.001 vs. T15; °°° *p* < 0.001 vs. T0. T0: baseline; T15: evaluation after 15 days from the start of therapy; T60: evaluation at the end of therapy.

**Table 4:** Statistical analysis obtained using a Bonferroni post-hoc test on the factors triggering the onset of vertigo.

**T0 Vs. T15**				
**Symptoms**	**Mean Difference**	***t***	***p* Value**	**Summary**
Instability	1.280	10.07	*p* < 0.001	°°°
Staggering	0.760	5.977	*p* < 0.001	°°°
Tendency to Fall	1.480	11.64	*p* < 0.001	°°°
Heaving	0.7600	5.977	*p* < 0.001	°°°
Position	0.6400	5.033	*p* < 0.01	°°
Bend Down	1.080	8.493	*p* < 0.001	°°°
March	1.160	9.122	*p* < 0.001	°°°
Head Movement	0.7200	5.662	*p* < 0.001	°°°
**T15 Vs. T60**				
**Symptoms**	**Mean Difference**	***t***	***p* Value**	**Summary**
Instability	0.4800	3.775	*p* < 0.05	*
Staggering	0.2400	1.887	*p* > 0.05	ns
Tendency to Fall	0.0200	0.1573	*p* > 0.05	ns
Heaving	0.000	0.000	*p* > 0.05	ns
Position	0.240	1.887	*p* > 0.05	ns
Bend Down	−0.08000	0.6291	*p* > 0.05	ns
March	0.08000	0.6390	*p* > 0.05	ns
Head Movement	0.2000	1.573	*p* > 0.05	ns

Data refer to n = 50 female patients. * *p* < 0.05 vs. T15; °° *p* < 0.001, °°° *p* < 0.001 vs. T0. T0: baseline; T15: evaluation after 15 days from the start of therapy; T-60: evaluation at the end of therapy.

## Data Availability

The data is not publicly available due to privacy restrictions.
